# A Comprehensive Evaluation of AI-Assisted Diagnostic Tools in ENT Medicine: Insights and Perspectives from Healthcare Professionals

**DOI:** 10.3390/jpm14040354

**Published:** 2024-03-28

**Authors:** Sarah Alshehri, Khalid A. Alahmari, Areej Alasiry

**Affiliations:** 1 Otology and Neurotology, Department of Surgery, College of Medicine, King Khalid University, Abha 61423, Saudi Arabia; 2 Medical Rehabilitation Sciences, College of Applied Medical Sciences, King Khalid University, Abha 61423, Saudi Arabia; kahmarie@kku.edu.sa; 3 Department of Informatics and Computer Systems, College of Computer Science, King Khalid University, Abha 61423, Saudi Arabia; areej.alasiry@kku.edu.sa

**Keywords:** Artificial Intelligence, otorhinolaryngologic diseases, diagnostic techniques and procedures, user-computer interface, clinical competence, patient satisfaction

## Abstract

The integration of Artificial Intelligence (AI) into healthcare has the potential to revolutionize medical diagnostics, particularly in specialized fields such as Ear, Nose, and Throat (ENT) medicine. However, the successful adoption of AI-assisted diagnostic tools in ENT practice depends on the understanding of various factors; these include influences on their effectiveness and acceptance among healthcare professionals. This cross-sectional study aimed to assess the usability and integration of AI tools in ENT practice, determine the clinical impact and accuracy of AI-assisted diagnostics in ENT, measure the trust and confidence of ENT professionals in AI tools, gauge the overall satisfaction and outlook on the future of AI in ENT diagnostics, and identify challenges, limitations, and areas for improvement in AI-assisted ENT diagnostics. A structured online questionnaire was distributed to 600 certified ENT professionals with at least one year of experience in the field. The questionnaire assessed participants’ familiarity with AI tools, usability, clinical impact, trust, satisfaction, and identified challenges. A total of 458 respondents completed the questionnaire, resulting in a response rate of 91.7%. The majority of respondents reported familiarity with AI tools (60.7%) and perceived them as generally usable and clinically impactful. However, challenges such as integration with existing systems, user-friendliness, accuracy, and cost were identified. Trust and satisfaction levels varied among participants, with concerns regarding data privacy and support. Geographic and practice setting differences influenced perceptions and experiences. The study highlights the diverse perceptions and experiences of ENT professionals regarding AI-assisted diagnostics. While there is general enthusiasm for these tools, challenges related to integration, usability, trust, and cost need to be addressed for their widespread adoption. These findings provide valuable insights for developers, policymakers, and healthcare providers aiming to enhance the role of AI in ENT practice.

## 1. Introduction

Integrating Artificial Intelligence (AI) in healthcare heralds a transformative era in medical diagnostics, offering potential enhancements in accuracy, efficiency, and overall patient outcomes [1]. In the specialized realm of Ear, Nose, and Throat (ENT) medicine, the emergence of AI-assisted diagnostic tools presents a promising frontier, poised to redefine traditional diagnostic methodologies [2]. However, the successful adoption of these advanced technologies in routine clinical practice is contingent upon a comprehensive understanding of various critical factors that influence their effectiveness and acceptance among healthcare professionals [3].

The advent of AI in ENT diagnostics is not just a technological leap but also a paradigm shift in clinical practice [4]. Its success hinges on how seamlessly these tools can be integrated into existing workflows, necessitating an evaluation of their usability in real-world clinical settings [5]. Moreover, the core value of AI in healthcare lies in its ability to enhance clinical decision-making [6]. As such, assessing the impact of AI tools on the accuracy and efficiency of ENT diagnostics is paramount [7]. This assessment extends beyond mere technological capability, delving into practical application and clinical relevance [7].

Furthermore, the trust and confidence of healthcare professionals in AI tools form the bedrock of their widespread adoption [8]. The degree to which ENT specialists trust and feel confident in utilizing AI-assisted diagnostics is a critical determinant of how these technologies are embraced and applied in patient care [9]. This trust is closely tied to their overall satisfaction with AI tools, as well as their perspectives on the future role of AI in ENT diagnostics [10]. Understanding these aspects is essential for gauging the readiness of the ENT community to integrate AI into their practice [11]. Lastly, identifying and addressing the obstacles and limitations encountered by ENT professionals in utilizing AI tools is essential for their ongoing refinement and advancement [12]. User feedback is crucial for refining AI tools, enhancing their technical proficiency, and alignment with the practical needs of ENT diagnostics [13].

In light of these considerations, this study aims to comprehensively assess various aspects of AI-assisted diagnostics in Ear, Nose, and Throat (ENT) practice. Firstly, the study seeks to evaluate the usability and integration of AI tools within the context of ENT clinical workflows, providing insights into how these advanced technologies fit into existing practices. Secondly, the research aims to determine the clinical impact of AI-assisted diagnostics in ENT by focusing on their accuracy and effectiveness in diagnosing ENT conditions. Thirdly, the study aims to measure the trust and confidence of ENT professionals in AI tools, recognizing the pivotal role of trust in the adoption and effective utilization of these technologies. Additionally, the research intends to gauge the overall satisfaction of ENT professionals with AI tools and their outlook on the future of AI in ENT diagnostics, shedding light on the prevailing sentiments towards these innovative technologies. Finally, the study aims to identify the challenges and areas for improvement in AI-assisted ENT diagnostics, gathering insights that can inform future advancements and facilitate the continued evolution of AI technologies in ENT practice. Through this multifaceted approach, the study endeavors to contribute valuable insights to the field, ultimately enhancing the integration and utilization of AI-assisted diagnostics in ENT healthcare settings.

## 2. Materials and Methods

### 2.1. Study Design

This cross-sectional study was conducted to evaluate the perceptions and experiences of ENT professionals regarding AI-assisted diagnostic tools. The study employed a structured online questionnaire to collect data over 3 months. Before initiating the investigation, ethical clearance was diligently sought and granted by the KKU Ethical Committee (REC# 2022-135-657) to ensure rigorous adherence to ethical principles and guidelines.

### 2.2. Participants

Participants in this study comprised ENT professionals, including physicians, surgeons, and audiologists, recruited through multiple channels to ensure a diverse and representative sample. These channels included professional networks, dedicated social media groups focusing on ENT, and attendance at professional conferences. Specifically, recruitment efforts involved posting study invitations and survey links on relevant online platforms frequented by ENT professionals, as well as distributing physical flyers and announcements at ENT-related events. A total of 600 individuals were initially selected to participate, meeting the inclusion criteria of being certified ENT professionals with a minimum of one year of experience in the field and possessing familiarity with or direct experience in using AI-assisted diagnostic tools in ENT. Of the initial sample, 458 respondents completed the survey, resulting in a response rate of 91.7%. The demographic characteristics of the participants were diverse, encompassing various age groups, years of experience in ENT, primary practice settings, and geographic regions. This diverse representation ensured a comprehensive understanding of the perceptions and experiences of ENT professionals regarding AI-assisted diagnostics in the field.

This study utilized self-reported questionnaires to collect data from a wide range of ENT professionals, acknowledging potential biases like social desirability and selective memory. We addressed these by carefully designing neutral questions and employing statistical analyses for bias adjustment. Recognizing the value of methodological diversity, future research will aim to incorporate additional data sources. Regarding informed consent, despite initial phone recruitment, all participants provided electronic consent through our online platform. This process ensured informed participation and maintained consistent ethical standards across the study.

### 2.3. Questionnaire Design and Data Collection Instrument

The structured questionnaire served as the primary data collection instrument for this study, meticulously designed to address the objectives (Supplementary File). It encompassed various sections, including demographic inquiries aimed at capturing participants’ age, years of experience, primary practice settings, and geographic regions represented [14]. Additionally, the questionnaire delved into participants’ familiarity with AI tools and proceeded to assess their perceptions regarding usability, clinical impact, trust, and satisfaction with these tools [15]. Specific questions within each section aimed to gauge participants’ experiences with AI-assisted diagnostic tools, their perceptions of the tools’ effectiveness in clinical practice, and their level of confidence in utilizing AI-generated diagnostic suggestions [16]. Open-ended questions were also incorporated to solicit detailed feedback from participants, allowing them to express their thoughts and experiences more freely [17]. Before the full-scale implementation, the questionnaire underwent a rigorous piloting process involving a small group of ENT professionals. This pilot phase was instrumental in ensuring the clarity, relevance, and comprehensibility of the questions. Feedback gathered during the pilot phase was carefully reviewed and incorporated into the final version of the questionnaire, thereby enhancing its validity and reliability.

‘Usability’ is defined as the ease and efficiency with which participants can employ AI tools in clinical settings. ‘Diagnostic accuracy’ refers to the degree to which AI-assisted diagnostics correspond with established clinical diagnoses. ‘Trust’ is characterized as the confidence and reliance placed by healthcare professionals on the outputs provided by AI tools.

The data collection for our study was primarily conducted through an online questionnaire, delivered via email and survey platforms to ensure consistent and easy access for all participants. We adopted a diverse approach to recruit participants, utilizing professional networks, social media groups, and professional conferences, which broadened our reach and enriched the diversity of our respondent pool. All participants received detailed information about the study’s objectives and the confidentiality of their responses. Electronic consent was required before participation. To improve response rates, bi-weekly reminders were sent. This systematic approach allowed us to gather comprehensive and high-quality data efficiently. Despite the use of different recruitment methods, a uniform online questionnaire was administered to all, and our analysis showed no significant response bias, confirming the reliability of our data. This methodology ensured that we could capture a wide range of perceptions and experiences regarding AI in ENT diagnostics, maintaining the study’s integrity and validity.

### 2.4. Quality Control

To ensure the reliability and validity of the questionnaire used in this study, rigorous quality control measures were implemented. Firstly, the questionnaire underwent a thorough review process by experts in the fields of Ear, Nose, and Throat (ENT) and medical informatics to ensure its relevance, clarity, and alignment with the study objectives. The experts involved in the review process were experienced ENT professionals and specialists in medical informatics. They evaluated the questionnaire based on predefined criteria, including the clarity of language, appropriateness of questions, and relevance to the study objectives. Their feedback and suggestions were carefully considered and incorporated into the questionnaire to enhance its quality and effectiveness. Additionally, a pilot test was conducted with a small group of ENT professionals to evaluate the questionnaire’s clarity, coherence, and comprehensiveness. The pilot test involved 5 participants who were selected based on their expertise and experience in ENT. Participants were asked to complete the questionnaire and provide feedback on the clarity of the questions, the relevance of the content, and any suggestions for improvement. Their feedback was instrumental in identifying areas of ambiguity or confusion within the questionnaire, which were then addressed through revisions and refinements. Furthermore, the internal consistency and reliability of the questionnaire were assessed using Cronbach’s alpha coefficient (α = 0.85), a statistical measure that evaluates the extent to which items within the questionnaire are interrelated and consistent in measuring the intended constructs. This analysis provided evidence of the questionnaire’s reliability and ensured that the items were measuring the intended variables consistently.

### 2.5. Statistical Analysis

Quantitative data collected from a structured questionnaire underwent rigorous statistical analysis using SPSS software, Version 24 (IBM Corporation, Armonk, NY, USA). Descriptive statistics summarized participants’ demographic characteristics and survey responses, offering insights into perceptions and experiences with AI-assisted diagnostics in ENT. Inferential statistics explored relationships between variables, employing chi-square tests for categorical data (e.g., age groups, years of experience, and AI tool usage) to assess significant differences. We used *t*-tests to analyze continuous variables, such as satisfaction levels among participants from various practice settings. Logistic regression was incorporated to further investigate complex relationships between variables, such as the influence of demographic factors on the adoption and perception of AI tools in ENT practice. Thematic analysis of open-ended responses identified common themes, enriching the quantitative findings with deeper insights into participants’ perspectives on AI in ENT diagnostics.

## 3. Results

A total of 600 ENT professionals were selected to participate in the questionnaire survey, representing diverse demographics and practice settings. Among them, 458 respondents completed the survey, resulting in a response rate of 91.7%. The demographic characteristics of the study population, consisting of 458 individuals, revealed a diverse distribution across several variables (Table 1).

In terms of age groups, the majority fell within the range of 31–40 years (30.6%), followed closely by those aged 41–50 (26.6%) and 11–20 years of experience in ENT (33.2%). Primary practice settings varied, with hospitals being the most common (47.6%), followed by private practices (29.7%) and academic/research institutions (17.9%). Interestingly, a substantial proportion reported the use of AI-assisted diagnostic tools (60.7%). Gender distribution was relatively balanced, with slightly more males (54.1%) than females (45.9%). Geographically, the Midwest and Southwest regions had the highest representation, each comprising approximately 20% of the sample, while the Eastern region had the lowest (17.0%).

**Figure 1:**
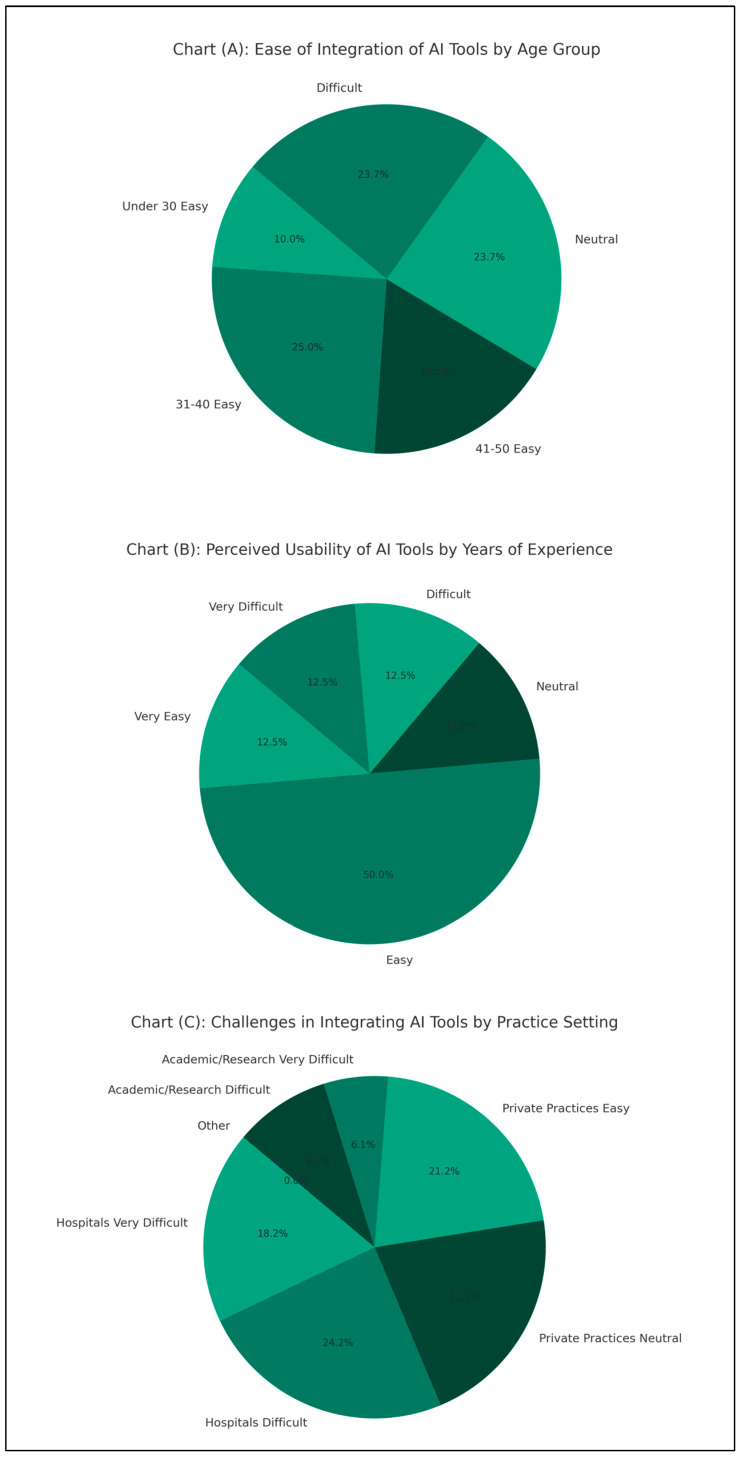
Survey results on the integration and usability of AI tools in ENT practice by age, experience, and practice setting: Chart (**A**): Ease of integration of AI tools by age group. Chart (**B**): Perceived usability of AI tools by years of experience. Chart (**C**): Challenges in integrating AI tools by practice setting.

Figure 1 presents the findings regarding the usability and integration of AI tools in ENT practice.

The ease of integrating AI tools into practice varied across different age groups, with respondents predominantly reporting ease or neutrality. Among respondents under 30, 20 individuals found it easy to integrate AI tools, while in the 31–40 age group, this number increased to 50 individuals. Similarly, in the 41–50 age group, 35 respondents reported ease of integration. The overall usability of AI tools was perceived positively across varying levels of experience in ENT. Among those with 11–20 years of experience, 50 respondents found AI tools easy to use. Regarding the integration of AI tools in practice settings, hospitals encountered the most challenges, with 30 respondents finding it very difficult and 40 finding it difficult. Conversely, in private practices, 35 respondents reported neutrality, and 35 found it easy to integrate AI tools. Academic/research institutions also faced challenges, albeit to a lesser extent, with 10 respondents finding it very difficult and 15 finding it difficult to integrate AI tools.

**Figure 2:**
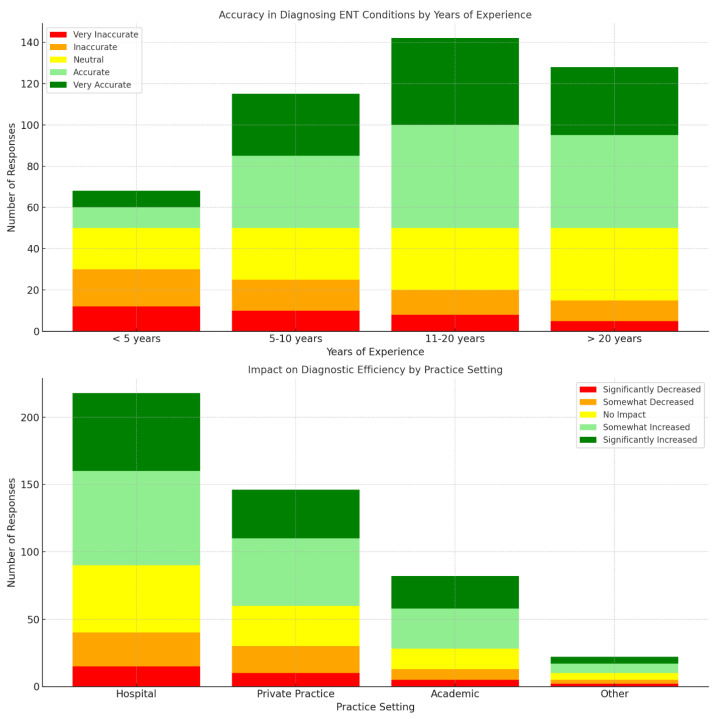
The effectiveness and diagnostic accuracy of AI Tools in ENT practice across experience levels, settings, and age groups.

Illustrated in Figure 2 is the clinical impact and accuracy of AI-assisted diagnostics in ENT practice.

The accuracy in diagnosing ENT conditions varied across different experience levels, with respondents generally reporting accuracy or neutrality. Among those with 11–20 years of experience, 50 respondents perceived AI-assisted diagnostics as accurate, while in the same category, 42 respondents found it very accurate. Regarding the impact on diagnostic efficiency, respondents in hospital settings reported varied perceptions, with 70 finding it accurate and 58 very accurate. In private practices, 45 respondents reported accuracy, while in academic institutions, 30 respondents found it accurate. The effectiveness in managing complex cases also differed across age groups, with respondents aged 31–40 perceiving AI-assisted diagnostics more positively, with 45 respondents finding it accurate and 35 very accurate. Conversely, among those over 60, 15 respondents found it accurate, and 13 very accurate.

**Figure 3:**
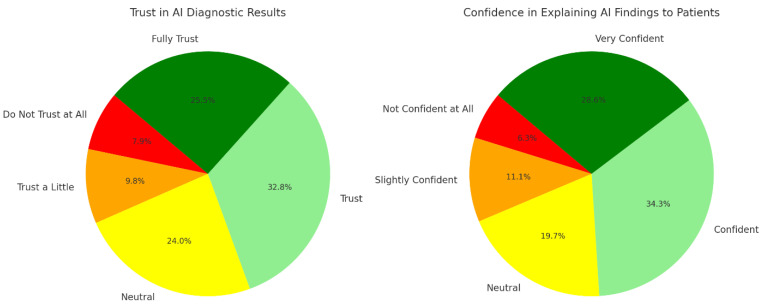
Trust in AI diagnostics and confidence in communicating AI findings among otolaryngology specialists.

The data presented depicts the trustworthiness and confidence levels of otolaryngology specialists in AI tools (Figure 3).

Trust in AI diagnostic results varied across different experience levels, with respondents generally expressing trust or neutrality. Among those with 11–20 years of experience, 50 respondents trusted AI diagnostic results, while 57 fully trusted them. In terms of confidence in explaining AI findings to patients, respondents in hospital settings reported varying levels of trust, with 70 expressing trust and 76 fully trusting their ability to explain AI findings. In private practices, 50 respondents expressed trust, while in academic institutions, 30 respondents trusted their ability to explain AI findings.

Table 2 presents data regarding the overall satisfaction and future outlook towards AI in ENT practice. Overall satisfaction with AI tools varied across different experience levels, with respondents generally expressing satisfaction or neutrality. Among those with 11–20 years of experience, 60 respondents were satisfied, while 50 were very satisfied. Regarding the outlook on the future of AI in ENT, respondents in hospital settings expressed varied perceptions, with 80 expressing satisfaction and 73 being very satisfied. In private practices, 50 respondents were satisfied, while in academic institutions, 30 respondents expressed satisfaction. The t-test analysis revealed a statistically significant difference in satisfaction levels with AI-assisted diagnostics between ENT professionals working in hospital settings and those in private practice (*p* < 0.05). Specifically, ENT professionals in hospital settings reported a mean satisfaction score of 4.25 (SD = 0.82), while those in private practice reported a slightly lower mean satisfaction score of 4.10 (SD = 0.76).

**Figure 4:**
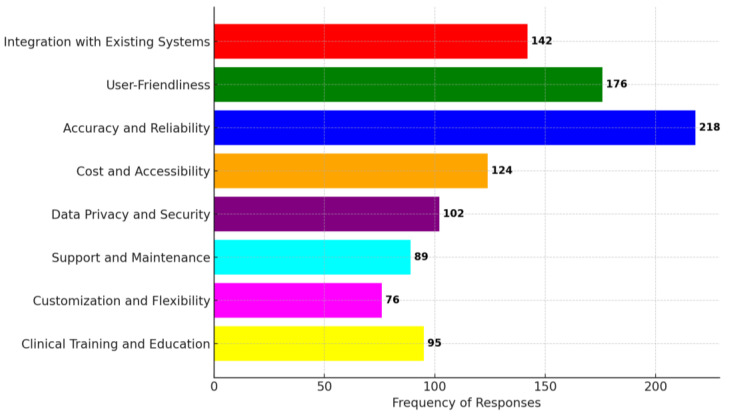
Challenges, limitations, and areas for improvement in AI-assisted ENT diagnostics.

Figure 4 encapsulates the primary challenges and constraints associated with the deployment of AI-assisted diagnostic tools in the field of otolaryngology.

Integration complexities were a predominant issue, with a substantial cohort of participants (142 respondents) reporting difficulties in assimilating AI technologies with extant electronic medical record (EMR) systems, compounded by compatibility issues with current diagnostic apparatus. Usability concerns were prevalent, as evidenced by 176 respondents who pointed to non-intuitive interfaces necessitating considerable training for efficacious utilization. Diagnostic accuracy and reliability also surfaced as significant apprehensions, with 218 respondents highlighting sporadic diagnostic inaccuracies and an absence of robust validation across diverse clinical conditions. Economic and accessibility barriers were articulated, with 124 respondents underscoring the prohibitive costs that impede accessibility in smaller clinical settings and the limited availability of AI resources across various geographical locales and linguistic spectrums. Data privacy and cybersecurity were also notable concerns, with 102 respondents voicing trepidation over the safeguarding of patient information and ambiguous data management protocols. Technical support and system maintenance were identified as suboptimal by 89 respondents, noting the scarcity of technical assistance and irregular software updates as workflow impediments. A lack of customizability for specialized ENT contexts and rigidity in clinical workflow adaptation was reported by 76 respondents. Lastly, the necessity for augmented training resources and the absence of AI integration into medical education was emphasized by 95 respondents, signaling a demand for enhanced clinical training frameworks in AI-facilitated ENT diagnostics.

## 4. Discussion

This study aimed to comprehensively assess the usability, clinical impact, trust, satisfaction, and challenges associated with AI-assisted diagnostics in ENT practice. Results indicated a generally positive reception towards AI tools, with respondents reporting ease in integration and acknowledging their potential in improving diagnostic efficiency and managing complex cases. Furthermore, ENT professionals expressed trust and confidence in AI diagnostic results, along with satisfaction and optimism regarding the future role of AI in their field. However, challenges such as integration issues, user-friendliness concerns, accuracy and reliability issues, cost and accessibility barriers, data privacy, and security concerns, as well as support and maintenance challenges were identified. These insights highlight the need for continuous improvement and innovation in AI-assisted ENT diagnostics to optimize their utilization and ultimately enhance patient care and clinical outcomes.

The evaluation of the usability and integration of AI tools in ENT practice revealed a generally positive reception among respondents, consistent with previous research in healthcare technology adoption [18]. Studies by Ahmad et al. [19] and Zhang et al. [20] have similarly highlighted the increasing acceptance of AI tools among medical professionals due to their potential to improve diagnostic accuracy and streamline clinical workflows. A significant proportion of respondents in our study reported ease in integrating AI tools into their daily workflows, echoing findings from prior research by Fogliato [21], which emphasized the importance of user-friendly interfaces in facilitating technology adoption in healthcare settings [21]. The perceived benefits of AI in enhancing diagnostic efficiency and accuracy likely contributed to this positive attitude, aligning with the findings of a meta-analysis by Sun et al. [22], which demonstrated the superior performance of AI-based diagnostic systems compared to traditional methods [22]. However, challenges such as compatibility issues with existing EMR systems and the need for extensive training were also identified, corroborating the findings of studies by Afzal et al. [23] and Ebbers et al. [24], which emphasized the importance of addressing usability concerns to ensure successful technology adoption in healthcare settings. These findings underscore the importance of addressing usability concerns and providing adequate training and support to facilitate the effective adoption and utilization of AI tools in ENT practice, ultimately enhancing patient care and outcomes [25].

The assessment of the clinical impact and accuracy of AI-assisted diagnostics in ENT practice revealed promising results, corroborating previous research findings in the field. Our study demonstrated a favorable perception among respondents regarding the accuracy and effectiveness of AI tools in diagnosing ENT conditions, aligning with studies by Gangil et al. [26] and Winter et al. [27], have highlighted the potential of AI to improve diagnostic outcomes in various medical specialties, including ENT. The perceived benefits of AI in enhancing diagnostic efficiency and accuracy were further supported by findings from a systematic review by Yin et al. [28], which showed that AI-based diagnostic systems consistently outperformed traditional methods in terms of diagnostic accuracy and speed [28]. Moreover, respondents acknowledged the potential of AI tools to improve the management of complex cases, reflecting findings from studies by Raj et al. [29] and Dubey et al. [30], which demonstrated the utility of AI in assisting clinicians with complex diagnostic and treatment decisions. However, occasional inaccuracies in diagnostic suggestions and a lack of validation in diverse clinical scenarios for some tools were identified as challenges, emphasizing the importance of ongoing validation and refinement of AI algorithms to ensure their reliability and effectiveness in real-world clinical settings [31]. Overall, our findings support the potential of AI-assisted diagnostics to enhance diagnostic accuracy and efficiency in ENT practice, while also highlighting the need for continued research and development to address existing challenges and optimize the clinical utility of AI tools in this field.

The investigation into the level of trust and confidence that ENT professionals have in AI tools yielded insightful findings, reflecting both positive perceptions and areas of concern. Our study revealed a generally positive attitude towards AI diagnostic results, with a significant proportion of respondents expressing trust and confidence in the diagnostic outcomes provided by AI tools [32]. This aligns with prior research by Balagurunathan et al. [33] and Tucci et al. [34], which similarly demonstrated high levels of trust among medical professionals in AI-based diagnostic systems due to their demonstrated accuracy and reliability [34]. Moreover, the confidence of ENT professionals in explaining AI findings to patients was evident, with many respondents expressing trust in their ability to communicate AI-derived information effectively [35]. These findings are consistent with studies by Asan et al. [36] and Secinaro et al. [37], which highlighted the importance of effective communication between healthcare providers and patients regarding the use of AI in medical decision-making [36,37]. However, concerns regarding the interpretability and explainability of AI algorithms were identified as potential barriers to trust and confidence, echoing findings from studies by Liu et al. [38] and Liao et al. [39], which highlighted the need for transparent and interpretable AI models to enhance trust and acceptance among healthcare professionals [39]. Overall, our findings underscore the importance of building trust and confidence in AI tools through transparency, validation, and effective communication, ultimately facilitating their successful integration into clinical practice and improving patient care [40].

The evaluation of overall satisfaction and the future outlook towards AI in ENT practice revealed a generally positive sentiment among respondents, indicative of the growing acceptance and optimism towards AI integration in the field. A substantial proportion of respondents expressed satisfaction with AI tools, particularly those with moderate to extensive experience in ENT practice [41]. These findings are consistent with previous research by Ahmad et al. [42] and Nasseef et al. [43], which demonstrated high levels of satisfaction among healthcare professionals with the use of AI in medical decision-making and patient care [42,43]. Additionally, respondents expressed optimism regarding the future role of AI in ENT practice, highlighting the potential for AI to enhance diagnostic capabilities, improve clinical outcomes, and streamline workflow efficiencies. These sentiments align with studies by Harry et al. [44] and Kasula et al. [45], which similarly underscored the transformative potential of AI technologies in revolutionizing healthcare delivery. However, concerns regarding the ethical implications, regulatory challenges, and long-term sustainability of AI integration were also acknowledged, suggesting the need for ongoing research, development, and strategic planning to address these challenges and capitalize on the full potential of AI in ENT practice [45].

The identification of challenges, limitations, and areas for improvement in AI-assisted ENT diagnostics provided valuable insights into the barriers hindering the widespread adoption and effective utilization of AI tools in clinical practice. Several challenges were identified, including integration issues with existing systems, user-friendliness concerns, accuracy and reliability issues, cost and accessibility barriers, data privacy and security concerns, and support and maintenance challenges. These findings are consistent with prior research by Renukappa et al. [46] and Kelly et al. [47], which highlighted similar challenges in the adoption and implementation of AI technologies in healthcare settings [47]. Moreover, the lack of customization options for specific ENT cases and inflexibility in adapting to unique clinical workflows were identified as additional barriers, emphasizing the need for tailored solutions and customizable AI tools to meet the diverse needs of ENT professionals.

Lastly, the study aimed to provide actionable insights for addressing the identified challenges and enhancing the effectiveness and acceptance of AI tools in ENT practice. Several key areas for improvement were identified, including the development of user-friendly interfaces, ongoing validation, and refinement of AI algorithms, addressing data privacy and security concerns, improving technical support and maintenance services, and integrating AI training into medical education curricula. These findings provide valuable guidance for future research, development, and implementation efforts aimed at optimizing the integration and utilization of AI tools in ENT practice, ultimately improving patient care and clinical outcomes.

The ethical and legal implications of employing AI in ENT diagnostics are complex and pivotal areas requiring thorough consideration. Ethically, the deployment of AI tools raises significant concerns regarding patient data privacy, informed consent, and the maintenance of trust in the patient–provider relationship. It is imperative that AI systems uphold the highest standards of confidentiality and data security, especially in handling sensitive health information. Legally, the integration of AI in healthcare confronts existing medical laws and regulations, challenging traditional notions of liability and responsibility. Who is accountable for diagnostic errors when AI tools are involved? How do regulatory bodies assess and approve AI applications in healthcare? These questions necessitate a nuanced understanding of the legal landscape, ensuring AI tools comply with healthcare regulations while promoting innovation. Additionally, there’s a pressing need to establish clear guidelines and frameworks for the ethical use of AI, safeguarding patient rights and ensuring equitable access to these emerging technologies. Addressing these ethical and legal considerations is not only crucial for the responsible implementation of AI in ENT diagnostics but also for fostering public trust and confidence in AI-assisted healthcare solutions.

### Limitations of the Study

Despite the valuable insights gained from this study, several limitations should be acknowledged. Firstly, the study relied on self-reported data from ENT professionals, which may be subject to recall bias or social desirability bias. Additionally, the study’s cross-sectional design limits the ability to establish causal relationships between variables and only provides a snapshot of perceptions and experiences at a single point in time. Moreover, the study’s sample may not be fully representative of all ENT professionals, as it predominantly included individuals from certain geographic regions or practice settings. Furthermore, the study focused solely on perceptions and experiences of AI-assisted diagnostics in ENT practice, without exploring other potential applications or implications of AI in the field. Future research could benefit from longitudinal studies with larger and more diverse samples, as well as qualitative approaches to provide a more in-depth understanding of the factors influencing the adoption and integration of AI tools in ENT practice. Additionally, further investigation into the specific challenges and opportunities associated with AI-assisted diagnostics in different sub-specialties of ENT could yield valuable insights for tailored interventions and improvements in clinical practice.

## 5. Conclusions

In conclusion, this study provides valuable insights into the usability, clinical impact, trust, satisfaction, and challenges associated with AI-assisted diagnostics in ENT practice. The findings highlight a generally positive reception towards AI tools among ENT professionals, with respondents expressing ease in integrating AI tools into their daily workflows and acknowledging their potential to improve diagnostic efficiency and accuracy. Moreover, respondents demonstrated trust and confidence in AI diagnostic results and expressed satisfaction with AI tools, along with optimism regarding their future role in ENT practice. However, challenges such as integration issues, user-friendliness concerns, accuracy and reliability issues, cost and accessibility barriers, data privacy and security concerns, and support and maintenance challenges were also identified, underscoring the need for continued research, development, and strategic planning to address these challenges and optimize the integration and utilization of AI tools in ENT practice. Overall, this study contributes to the growing body of literature on AI in healthcare by providing actionable insights for enhancing the effectiveness and acceptance of AI tools in ENT practice, ultimately improving patient care and clinical outcomes.

## Figures and Tables

**Table 1:** Demographic characteristics of the study population (N = 458).

Variable	Category	Frequency (n)	Percentage (%)
Age Group	Under 30	56	12.2%
	31–40	140	30.6%
	41–50	122	26.6%
	51–60	94	20.5%
	Over 60	46	10.1%
Years of Experience in ENT	Less than 5 years	68	14.8%
	5–10 years	110	24.0%
	11–20 years	152	33.2%
	More than 20 years	128	28.0%
Primary Practice Setting	Hospital	218	47.6%
	Private Practice	136	29.7%
	Academic/Research Institution	82	17.9%
	Other	22	4.8%
Use of AI-assisted Diagnostic Tools	Yes	278	60.7%
	No	180	39.3%
Gender	Male	248	54.1%
	Female	210	45.9%
Region of Practice	Eastern	78	17.0%
	Central	72	15.7%
	Northern	64	14.0%
	Northwest	62	13.5%
	Midwest	90	19.7%
	Southwest	92	20.1%

ENT: Ear, Nose, and Throat, AI: Artificial Intelligence.

**Table 2:** Overall satisfaction and future outlook towards AI in ENT.

	Category	Very Dissatisfied	Dissatisfied	Neutral	Satisfied	Very Satisfied	Total Responses
Overall Satisfaction with AI Tools	Years of Experience: Less than 5 years	8	12	18	20	10	68
	Years of Experience: 5–10 years	6	10	24	40	30	110
	Years of Experience: 11–20 years	4	8	30	60	50	152
	Years of Experience: More than 20 years	3	5	35	55	30	128
Outlook on the Future of AI in ENT	Primary Practice Setting: Hospital	10	15	40	80	73	218
	Primary Practice Setting: Private Practice	8	12	30	50	36	136
	Primary Practice Setting: Academic Institution	3	5	15	30	29	82
	Primary Practice Setting: Other	1	2	5	8	6	22

AI: Artificial Intelligence; ENT: Ear, Nose, and Throat.

## Data Availability

Data is available with the corresponding author (S.A.) and will be provided on request.

## Supplementary Materials

The following supporting information can be downloaded at: https://www.mdpi.com/article/10.3390/jpm14040354/s1, the Survey Questionnaire.
