# Supplementary material for: A Comprehensive Evaluation of AI-Assisted Diagnostic Tools in ENT Medicine: Insights and Perspectives from Healthcare Professionals
Source: J Pers Med. 2024 Mar 28;14(4):354. doi: 10.3390/jpm14040354 (PMC11051468; doi:10.3390/jpm14040354)
Supplement: Supplementary file 1 [file jpm-14-00354-s001.zip › jpm-2914669-supplementary.pdf]

SURVEY QUESTIONNAIRE

Demographic Information

1. Age Group:
  - [ ] Under 30
  - [ ] 31-40
  - [ ] 41-50
  - [ ] 51-60
  - [ ] Over 60

2. Years of Experience in ENT:
  - [ ] Less than 5 years
  - [ ] 5-10 years
  - [ ] 11-20 years
  - [ ] More than 20 years

3. Primary Practice Setting:
  - [ ] Hospital
  - [ ] Private Practice
  - [ ] Academic/Research Institution
  - [ ] Other: [Please Specify]

4. Have you previously used any AI-assisted diagnostic tools in your practice?
  - [ ] Yes
  - [ ] No

Section 1: Familiarity and Perception

5. How familiar are you with the application of AI in ENT diagnostics?
  - [ ] Very unfamiliar
  - [ ] Unfamiliar
  - [ ] Neutral
  - [ ] Familiar
  - [ ] Very familiar

6. What is your perception of the potential impact of AI in ENT diagnostics?
  - [ ] Very negative
  - [ ] Negative
  - [ ] Neutral
  - [ ] Positive
  - [ ] Very positive

Section 2: Usability and Functionality

7. If you have used AI tools, how easy was it to integrate them into your routine practice?
  - [ ] Very difficult
  - [ ] Difficult

- [ ] Neutral
- [ ] Easy
- [ ] Very easy

8. How do you rate the user-friendliness of the AI tools you have used?
- [ ] Very poor
- [ ] Poor
- [ ] Neutral
- [ ] Good
- [ ] Excellent

Section 3: Clinical Impact

9. In your opinion, how accurately do AI tools assist in diagnosing ENT conditions?

- [ ] Very inaccurately
- [ ] Inaccurately
- [ ] Neutral
- [ ] Accurately
- [ ] Very accurately

10. Have AI-assisted tools impacted the efficiency of your diagnostic process?
- [ ] Significantly decreased efficiency
- [ ] Somewhat decreased efficiency
- [ ] No impact
- [ ] Somewhat increased efficiency
- [ ] Significantly increased efficiency

11. To what extent do you believe AI tools help in managing complex ENT cases?
- [ ] Not at all helpful
- [ ] Slightly helpful
- [ ] Moderately helpful
- [ ] Very helpful
- [ ] Extremely helpful

Section 4: Trust and Dependability

12. How much do you trust the results provided by AI diagnostic tools?
- [ ] Do not trust at all
- [ ] Trust a little
- [ ] Neutral
- [ ] Trust
- [ ] Fully trust

13. Do you feel confident explaining AI tool's diagnostic findings to your patients?

- [ ] Not confident at all
- [ ] Slightly confident
- [ ] Neutral
- [ ] Confident
- [ ] Very confident

Section 5: Overall Experience and Future Prospects
14. Overall, how satisfied are you with the current AI-assisted diagnostic tools for ENT?
- [ ] Very dissatisfied
- [ ] Dissatisfied
- [ ] Neutral
- [ ] Satisfied
- [ ] Very satisfied

15. Do you see a role for AI in the future of ENT diagnostics?
- [ ] Definitely not
- [ ] Probably not
- [ ] Not sure
- [ ] Probably yes
- [ ] Definitely yes

Section 6: Feedback and Suggestions
16. What are the primary challenges or limitations you have faced while using AI in ENT diagnostics?

Answer:

17. In your view, what improvements or enhancements are needed in AI tools to make them more effective for ENT diagnostics?

Answer:

18. Are there specific ENT conditions or areas where you believe AI tools could be particularly beneficial?

Answer:

19. Please share any additional comments or insights you have regarding the use of AI in ENT practice.

Answer:

Instructions for Participants:

- Please answer each question based on your personal experience and opinions.
- Your responses are confidential and will be used to improve AI-assisted diagnostic tools in the field of ENT.
- Thank you for your valuable contribution to advancing ENT care.

Conclusion

We appreciate your time and effort in completing this questionnaire. Your insights are vital for understanding the current status and future potential of AI in ENT diagnostics.
